# Chronic Right Ventricular Pacing Post-Transcatheter Aortic Valve Replacement Attenuates the Benefit on Left Ventricular Function

**DOI:** 10.3390/jcm13154553

**Published:** 2024-08-04

**Authors:** Chieh-Ju Chao, Deepa Mandale, Juan M. Farina, Merna Abdou, Pattara Rattanawong, Marlene Girardo, Pradyumma Agasthi, Chadi Ayoub, Mohammad Alkhouli, Mackram Eleid, F. David Fortuin, John P. Sweeney, Peter Pollak, Abdallah El Sabbagh, David R. Holmes, Reza Arsanjani, Tasneem Z. Naqvi

**Affiliations:** 1 Department of Cardiovascular Diseases, Mayo Clinic Arizona, 5777 East Mayo Blvd, Phoenix, AZ 85054, USA; chao.chiehju@mayo.edu (C.-J.C.); mandale.deepa@mayo.edu (D.M.); juan.farina@gmail.com (J.M.F.); rattanawong.pattara@mayo.edu (P.R.); pradyumna_agasthi@hotmail.com (P.A.); ayoub.chadi@mayo.edu (C.A.); fortuin.david@mayo.edu (F.D.F.); sweeney.john3@mayo.edu (J.P.S.); arsanjani.reza@mayo.edu (R.A.); 2 Department of Cardiovascular Diseases, Mayo Clinic Rochester, Rochester, MN 55902, USA; alkhouli.mohamad@mayo.edu (M.A.); eleid.mackram@mayo.edu (M.E.); holmes.david3@mayo.edu (D.R.H.); 3 Department of Medicine, Mayo Clinic Arizona, Scottsdale, AZ 85259, USA; abdou.merna@mayo.edu; 4 Department of Bioinformatics, Mayo Clinic Arizona, Scottsdale, AZ 85259, USA; girardo.marlene@mayo.edu; 5 Department of Cardiovascular Diseases, Mayo Clinic Jacksonville, Jacksonville, FL 32224, USA; pollak.peter@mayo.edu (P.P.); elsabagh.abdallah@mayo.edu (A.E.S.)

**Keywords:** transcatheter aortic valve implantation, pacemaker, left ventricular ejection fraction, LV longitudinal strain, right ventricular pacing, clinical outcome

## Abstract

**Background:** Conduction abnormality post-transcatheter aortic valve implantation (TAVI) remains clinically significant and usually requires chronic pacing. The effect of right ventricular (RV) pacing post-TAVI on clinical outcomes warrants further studies. **Methods:** We identified 147 consecutive patients who required chronic RV pacing after a successful TAVI procedure and propensity-matched these patients according to the Society of Thoracic Surgeons (STS) risk score to a control group of patients that did not require RV pacing post-TAVI. We evaluated routine echocardiographic measurements and performed offline speckle-tracking strain analysis for the purpose of this study on transthoracic echocardiographic (TTE) images performed at 9 to 18 months post-TAVI. **Results:** The final study population comprised 294 patients (pacing group n = 147 and non-pacing group n = 147), with a mean age of 81 ± 7 years, 59% male; median follow-up was 354 days. There were more baseline conduction abnormalities in the pacing group compared to the non-pacing group (56.5% vs. 41.5%. *p* = 0.01). Eighty-eight patients (61.6%) in the pacing group required RV pacing due to atrioventricular (AV) conduction block post-TAVI. The mean RV pacing burden was 44% in the pacing group. Left ventricular ejection fraction (LVEF) was similar at follow-up in the pacing vs. non-pacing groups (57 ± 13.0%, 59 ± 11% *p* = 0.31); however, LV global longitudinal strain (−12.7 ± 3.5% vs. −18.8 ± 2.7%, *p* < 0.0001), LV apical strain (−12.9 ± 5.5% vs. 23.2 ± 9.2%, *p* < 0.0001), and mid-LV strain (−12.7 ± 4.6% vs. −18.7 ± 3.4%, *p* < 0.0001) were significantly worse in the pacing vs. non-pacing groups. **Conclusions:** Chronic RV pacing after the TAVI procedure is associated with subclinical LV systolic dysfunction within 1.5 years of follow-up.

## 1. Background/Introduction

The transcatheter aortic valve implantation (TAVI) procedure has rapidly increased in the past decade and has changed the landscape of managing aortic stenosis. In the period between 2012 and 2017, TAVI contributed to about 27% of all aortic valve replacement procedures in the United States [1]. As expected, TAVI patients were older and had a higher clinical risk than patients who underwent surgical aortic valve replacement (SAVR) [1]. Potential complications after the TAVI procedure include coronary artery obstructions/myocardial infarction peri-procedural stroke and the need for new permanent pacemaker implantation. The newer-generation valve design has led to a significant reduction in procedural complications; however, the rate of permanent pacemaker (PPM) implantation remains high [2]. Certain risk factors such as baseline conduction disturbance, shorter membranous septum length, and the non-coronary cusp calcium volume [3,4,5,6,7] have been identified as predictors of PPM implantation after TAVI.

The adverse effects of chronic right ventricular (RV) pacing have been demonstrated in prior studies in non-TAVI patient cohorts, including increased rates of atrial fibrillation, heart failure hospitalization, and all-cause mortality due to mechanical and electrical dyssynchrony [8,9,10]. However, the impact of chronic pacing in TAVI patients has been controversial in the available literature. In patients who had a new PPM post-TAVI, some studies have shown increased mortality or combined mortality/rehospitalization at one year [5]; however, the adverse effect of new PPM on mortality was not consistently observed in other studies [3,8,9,11]. Despite the controversy in hard clinical endpoints, studies have shown impaired left ventricular (LV) function in patients requiring a new PPM post-TAVI at 2-year follow-up [11,12], more marked in patients with reduced LV function at baseline and increased risk of heart failure hospitalization at longer follow up [12], but no increased risk of cardiac mortality or overall mortality after adjusting for baseline clinical characteristics. Importantly, many of the above TAVI studies were based on large-scale clinical registries, and detailed data about the pacemaker type, indication, and the most importantly, pacing burden were not available in most [3,5,8,11] studies, excepting some [12]. It is also possible that early stage pacing-induced cardiac dysfunction is subclinical, requiring more sensitive methods such as speckle strain echocardiography (STE) for detection [13,14,15].

We therefore designed this study to decipher the effects of chronic RV pacing on LV systolic function in patients who underwent TAVI. We hypothesized that chronic right ventricular pacing is associated with impaired LV systolic function in TAVI patients and can be detected by STE.

## 2. Methods

### 2.1. Study Design

This study was designed to compare the effect of chronic RV pacing post-TAVI procedure on clinical outcomes and LV systolic function. Given that TAVI patients are at high risk of comorbidity, we chose the STS risk score and other associated factors for propensity matching to achieve risk-balanced groups. The study group (pacing) was initially selected, followed by identifying the control (non-pacing) group through propensity matching to obtain relevant data.

### 2.2. Patient Cohort

This study was reviewed and approved by the Mayo Clinic IRB. In the period of 1 January 2012–31 December 2017, adult patients who underwent a successful transcatheter aortic valve replacement (TAVI) at Mayo Clinic sites (Rochester, Arizona, and Florida) and developed conduction system disturbance requiring new permanent pacemaker implantation were identified from the Mayo Clinic institutional National Cardiovascular Disease Registry (NCDR)-TAVI database. Patients who received a biventricular pacemaker/ICD and those with a prior pacemaker were excluded. We used 1:1 propensity score matching to estimate the marginal effect of a pacemaker on TAVI, accounting for covariates of age, sex, race, and STS risk. Specifically, a 1:1 nearest neighbor propensity score matching, using a caliper window of 0.1, without replacement with a propensity score was estimated using logistic regression of having a pacemaker on the covariates. The R package—MatchIt (https://www.r-project.org/) was used for this analysis.

To evaluate the effect of RV pacing burden, patients in the pacing group were further divided into the high-pacing burden group (HPB group, pacing ≥ 40%) and the low-pacing burden group (LPB group, pacing < 40%). This cutoff was chosen based on prior reports showing that ≥40% RV pacing burden is a risk factor for pacing-induced cardiomyopathy [10,16].

### 2.3. Two-Dimensional Transthoracic Echocardiography and Speckle-Tracking Strain Imaging

For each patient, baseline (pre-TAVI procedure) and follow-up (at 9–18 months post-TAVI) transthoracic echocardiography (TTE) studies were reviewed to obtain all echocardiographic measurements except for strain data. All measurements were performed according to the guidelines [17,18,19]. LVEF was measured by Simpson’s method. For both baseline and follow-up TTE, apical 2-, 3-, and 4-chamber views were exported for offline LV speckle-racking strain imaging analysis. The strain measurements were performed by independent observers (MA and DM) using commercial ECHOINSIGHT software (version 2.2.6.2230, Epsilon Imaging, Ann Arbor, MI, USA).

### 2.4. ECG/Pacemaker Data

Baseline ECG data was extracted from the NCDR-TAVI database. In patients who required a new pacemaker/implantable cardioverter-defibrillator (ICD) implantation, the interrogation data at one-year post-TAVI were reviewed to obtain information on pacing indication, pacing mode, RV pacing burden, the presence of tachyarrhythmia (atrial fibrillation, ventricular fibrillation, sustained ventricular tachycardia, and other tachyarrhythmias).

### 2.5. Clinical Outcomes

The clinical outcome data, including heart failure hospitalization (HFH) and all-cause mortality, were extracted from the NCDR-TAVI database. Each event of “valve-related readmission” and “non-valve-related readmission” charted in the database was reviewed and only counted as a HFH event if heart failure was documented in the discharge summary.

### 2.6. Statistical Analysis

Propensity-matched patients were divided into pacing and non-pacing (control) groups for comparison. Demographic information was summarized by mean and standard deviation, while categorical was summarized by count and percent. For the two groups, the Student *t*-test and Mann–Whitney rank test were used for comparing normally and non-normally distributed continuous variables, respectively. The chi-square test was used to compare the distribution of the categorical variables. Kaplan–Meier survival analysis was used to assess the difference in survival between the two groups, and a log-ranked test was used to assess significance. Cox regressions were used for associations to obtain hazard ratios (HRs) with 95% confidence intervals (CI) without and with adjustment for covariates. Left Ventricular Global Longitudinal Strain (LVGLS) > −10.0% was used as the cutoff of impaired LVGLS endpoint in logistic regression analysis. The primary endpoint of interest is LVGLS; secondary endpoints were all-cause mortality and HFH. All hypothesis tests were two-sided with a *p*-value < 0.05. All analysis was performed in SAS 9.4 (SAS Institute Inc., Cary, NC, USA).

## 3. Results

### 3.1. Patient Cohort

A total of 294 propensity-matched patients were included for the final analysis with 147 patients in each group. Supplemental Table S1 presents a summary of the four characteristics used for the propensity matching of both the matched and initial unmatched cohorts. The median follow-up time was 354 days (min 9 days, max 506 days). The mean age was 81.1 ± 7.4 years old, and 174 (59.2%) were male, 284 (96.6%) white, and the mean STS-risk score was 8.5 ± 6.9. There were more patients who had prior PCI (32.0% vs. 17.7%, *p* = 0.005) in the non-pacing group, although more patients in the pacing group had prior CABG. The pacing group had more NYHA class III/IV at baseline (82.3% vs. 72.8% *p* = 0.050) and a significantly higher rate of pre-TAVI conduction defect (56.5% vs. 41.5% vs. *p* = 0.010). Otherwise, there was no significant difference among the baseline demographics, medications, and labs including age, sex, race, STS risk score, prior MI, prior CABG, prior stroke, prior CAD, current dialysis, smoking, hypertension, diabetes, atrial fibrillation/atrial flutter, hemoglobin, creatinine, aspirin, ACE inhibitor, ARB, beta-blocker, and warfarin. Detailed data are summarized in Table 1.

### 3.2. ECG/Pacemaker Data

Within the pacing group, all of the patients received a pacemaker, and none of the pacing patients received biventricular pacing. The median RV pacing burden was 26.0% (interquartile range: 2.0–95%). In this group, 43.5% (n = 64) of patients had a pacing burden ≥ 40% and the median RV pacing burden was 96%. Pacing indication was predominantly AV block (39.9%) or complete AV block (21.7%), followed by LBBB (25.2%). Also, 83.1% (n = 123) patients were in DDD/DDDR mode, and 14.2% (n = 21) patients were in VVI/VVIR mode. Compared to the non-paced group, we observed significantly higher proportions of RBBB (29.9% vs. 17.7% *p* = 0.02), first-degree AV block (29.3% vs.18.4% *p* = 0.04), and left anterior fascicular block (15.0% vs. 4.8% *p* = 0.006) and longer QT (427.8 ± 44.6 ms vs. 415.6 ± 41.8 ms, *p* = 0.015) in the baseline ECG of the paced group. Detailed data are summarized in Table 1.

### 3.3. Two-Dimensional Transthoracic Echocardiography and Speckle-Tracking Strain Imaging

At baseline, there was no difference in the baseline LVEF (non-pacing vs. pacing: 53.8 ± 13.9% vs. 56.7 ± 13.0%, *p* = 0.455); however, the LVGLS was significantly different (−14.9 ± 4.3% vs. −13.6 ± 3.5%, *p* = 0.008). When comparing the follow-up and baseline measurements within each group, there was a significant improvement in the follow-up LVEF in the non-pacing group (*p* = 0.001) but not in the pacing group (*p* = 0.775). In addition, the non-pacing group showed an improvement in LV strain measurements (baseline vs. follow-up LVGLS: −14.9 ± 4.3% vs. −18.8 ± 2.7%, *p* < 0.0001). When comparing the follow-up data of the two groups, there was no significant difference in LVEF (non-pacing vs. pacing: 58.8 ± 11.2% vs. 57.1 ± 13.0%, *p* = 0.308), and the strain was significantly worse in the pacing group (LVGLS: −13.6 ± 3.5% vs. −12.7 ± 3.5%, *p* = 0.021). Strain analysis demonstrated significantly worse LV global longitudinal strain (−18.8 ± 2.7% vs. −12.7 ± 3.5%, *p* < 0.0001), LV apex (−23.2 ± 9.2% vs. −12.9 ± 5.5%, *p* < 0.0001), and mid (−18.7 ± 3.4% vs. −12.7 ± 4.6%, *p* < 0.0001) regional strain in the pacing group compared to the non-pacing group at follow up. We did not observe significant differences in diastolic function measurements (average E/e’, E/A ratio, RV systolic pressure) between the two groups. Detailed data are summarized in Table 2.

Effect of RV Pacing Burden: On subgroup analysis, the high-pacing burden (HPB, n = 64) subgroup (with RV pacing burden of more than 40%) had a trend for worse LVGLS (−12.0 ± 3.8% vs. −13.1 ± 3.3%, *p* = 0.059) compared to the low-pacing burden (LPB, n = 83) subgroup at follow-up. At a regional level, the strain was worse in the LV apical region (−11.4 ± 6.4% vs. −13.9 ± 4.6%, *p* = 0.0091) and mid-LV regional (−11.8 ± 5.4% vs.−13.4 ± 3.8%, *p* = 0.0419) but not at the LV basal region (−11.8 ± 4.5% vs. −12.1 ± 3.8%, *p* = 0.3611) in the high vs. low RV pacing burden group.

### 3.4. Clinical Outcomes

**Figure 1:**
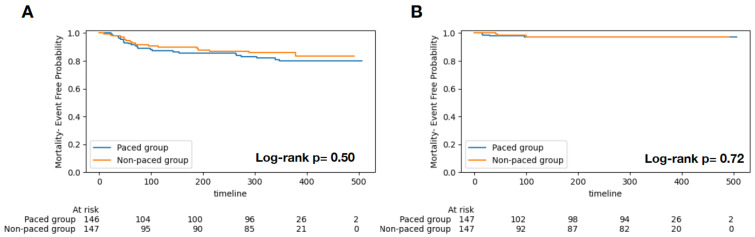
Kaplan–Meier survival curves of the two groups (pacing versus non-pacing). There was no significant prognostic difference for all-cause mortality ((**A**), *p* = 0.50) and heart failure hospitalization ((**B**), *p* = 0.72).

All-cause mortality occurred in 13.9% (n = 41) patients, and 2.4% of them (n = 7) had HFH events. The Kaplan–Meier analysis did not show significant differences in HFH (Figure 1A, log-rank *p* = 0.72) and mortality endpoints (Figure 1B, log-rank *p* = 0.50) between the paced and non-paced groups. The Cox regression model did not reveal significant associations between pacing and HFH (HR 1.32, 95% CI 0.29–5.95, *p* = 0.720) and mortality (HR 0.94, 95% CI 0.74–1.18, *p* = 0.573) after adjusting for age, sex, and STS risk score. In terms of NYHA class, a majority of patients had class III at baseline (63.9% vs. 70.7%). At the time of follow-up, patients were predominantly in NYHA class I in both groups (73.5% vs. 74.1%), and there was no significant difference between the two groups (*p* = 0.270) (Table 3).

Logistic regression showed that pacing burden is an independent predictor of compromised follow-up LVGLS (>−10.0%) in univariate (OR 1.01 per 1 unit increase, 95% CI: 1.00–1.02, *p* = 0.034) and multivariate analysis (OR 1.01 per 1 unit increase, 95%CI: 1.00–1.02, *p* = 0.040; adjusted for age and sex).

## 4. Discussion

The major findings of this study are as follows: (1) chronic RV pacing post-TAVI is associated with suboptimal improvement of LVEF and worse LV strain; (2) pre-existing RBBB, first-degree AV block, and LAFB are more prevalent in those who require chronic RV pacing post-TAVI; (3) RV pacing burden of >40% appears to confer worse adverse effect on LV strain post-TAVI; and (4) need for a pacemaker after a TAVI procedure is not associated with increased mortality or heart failure hospitalization at 1-year follow up, regardless of the pacing burden.

To the best of our knowledge, this is the first study that demonstrates the adverse effect of RV pacing detected by speckle-tracking strain despite having normal LVEF in TAVI patients. Our results support the use of speckle-tracking strain analysis in the post-procedural care of TAVI patients to detect early stage or subclinical LV dysfunction in this patient cohort.

### 4.1. Impact of Chronic Pacing on TAVI Patients

Prior studies have shown variable clinical outcome results in patients who required a new pacemaker after the TAVI procedure [3,5,8,11,12]. Potential explanations for these conflicting data are the relatively short follow-up duration and variable clinical presentations of pacemaker-induced cardiomyopathy (PiCM) [20,21,22]. Unfortunately, detailed pacemaker data were not available in prior large TAVI studies [3,5,8,11,12], and myocardial functional endpoints are only reported in some of them [11,12]. We attempt to address these controversies by evaluating pacing burden and evaluating LV function by speckle-tracking strain measurements besides LVEF in our study.

### 4.2. The Effect of Chronic RV Pacing on Clinical Outcomes

TAVI outcomes often rely on baseline comorbidities and the success of the procedure. The two groups had similar baseline characteristics except that the non-pacing group had a higher rate of prior PCI, more unplanned vascular surgery, and a higher post-TAVI mean gradient (Table 1). Our findings are comparable to published studies [9,11,23] suggesting that a new pacemaker after the TAVI procedure is not associated with increased all-cause mortality or HFH. The studies reported significance in clinical outcomes of increased mortality at 2 years [5] or increased HFH [12] at a median of 4-year follow-up. Our findings are likely due to the relatively shorter follow-up, normal baseline LVEF (only 14 patients had baseline LVEF < 50%), and lower overall pacing burden (median 26.0%) as well as lower STS score. Pacemaker dependency was similar to the data reported in prior studies [3,12,24]; our pacing group only had 43% of patients with an RV pacing burden of >40%. Our findings support that the pacing burden should be closely monitored post-TAVI and the pacing burden minimized, if clinically feasible. In Cox regression analysis, RV pacing burden was not associated with mortality or HFH. It is possible that the 1-year follow-up duration may not be long enough for the detrimental effects of chronic RV pacing to translate into clinical events in TAVI patients, even in patients with a high pacing burden. This is in line with prior observation that, in patients with normal baseline LVEF, it can take up to 4 to 5 years to develop PiCM [24], which also explains the findings in most of the TAVI studies with relatively shorter follow-up duration [9,11]. 

Of note, Fadahunsi et al. reported significantly higher STS-PROM scores in their PPM group, which essentially reflected a higher risk group at baseline, and this covariate was not adjusted in their multivariate analysis [5].

In contrast, studies have observed the adverse effect of post-TAVI pacing on soft clinical endpoints. Urena et al. did not observe a significant difference between all-cause mortality and the composite endpoint of all-cause mortality and HFH but instead a trend towards decreased LVEF [11]. In another study, post-TAVI PPM implantation was associated with worse short-term quality of life but had no impact on long-term adverse outcomes [8].

Importantly, the mean baseline LVEF in our study was about 55%, which was similar to the studies above [3,5,11,12]. In general pacemaker patient populations, a baseline LVEF of more than 50% is considered a protective factor for adverse events such as death or HFH [25]. Most TAVI patients have relatively good baseline LVEF, and that likely provides more reservoir for them to tolerate the adverse effect of chronic RV pacing.

In earlier studies, TAVI patients were sicker and with more underlying comorbidities [9], so it is possible that these patients may not survive long enough to develop the detrimental effects of chronic RV pacing. With the expanding of TAVI indications to lower-risk and younger populations [26], the long-term adverse effect of chronic RV pacing must be taken seriously.

### 4.3. The Effect of Chronic RV Pacing on LV Function

Normalization/improvement of LV systolic function is anticipated in patients who underwent a TAVI procedure, even in patients with low LVEF at baseline [11,12,27,28]. It is observed that patients who received PPM after TAVI may have either suboptimal LVEF evolution or less improvement when compared to the non-pacing cohort [11,12]. Urena et al. reported an initial improvement followed by down-trending LVEF at follow-up in patients who received a pacemaker post-TAVI, regardless of the type (single/dual chamber) of the pacemaker [11]. Chamandi et al. also reported “less improvement” of LVEF in pacing patients over their 4-year follow-up duration for patients with normal or low LVEF at baseline [12].

Our findings on LVEF changes are consistent with the above studies (pre- vs. post-TAVI): specifically, in the pacing group, there was no improvement in LVEF and slight worsening of LV strain whereas the non-pacing group had a significant improvement in LVEF and strain. This suggests that the hemodynamic benefits of aortic valve correction may be offset by RV pacing. Notably, both studies indicated that the negative effect of RV pacing on LVEF can persist subtly over extended follow-up periods, even with higher pacing burdens [12].

While strain analysis is reported to be a useful tool and a predictor of PiCM [12,13,27,29,30], its value has not been well explored in a TAVI-PPM patient cohort. We observed post-TAVI LV strain improvement in the non-pacing group, similar to prior studies [27,28]. In contrast, in the pacing group, besides unchanged LVEF, the mean LVGLS was significantly worse post-TAVI (−13.6 ± 3.5% vs. −12.7 ± 3.5%, *p* = 0.021). The head-to-head comparison of the follow-up strain showed a 32% difference between the two groups, further supporting the hypothesis that RV pacing offsets the benefit of TAVI. However, as data on ventricular rhythm status were not available during echo studies, the possibility that LVEF and LV strain may have been compromised by pacing or asynchrony cannot be excluded [29,31].

Furthermore, when compared to the non-pacing group, the regional longitudinal strain significantly worsened in the pacing group at the LV apex region (−17.1 ± 5.4% vs. −12.9 ± 5.5%, *p* < 0.0001) and at the LV mid-region (−18.7 ± 3.4 vs. −13.9 ± 5.0, *p* < 0.0001), but there was no difference at the LV basal region. This demonstrated that compromised strain function has a pacing-related effect since the mid and apex regions are the most common anatomical areas for RV lead placement [30]. A similar LV strain pattern was observed in the pacing burden subgroup analysis. Comparing the HBP groups to the LBP group, we observed a trend for worse LVGLS (−12.0 ± 3.8% vs. −13.1 ± 3.3%, *p* = 0.060) and significantly worse apex (−11.4 ± 6.4% vs. −13.1 ± 3.3%, *p* = 0.009) and mid (−11.8 ± 5.4% vs. −13.4 ± 3.8%, *p* = 0.042) LV regional strain. In addition, multivariate logistic regression showed that pacing burden was an independent predictor of compromised follow-up LV GLS (>−10.0%). These findings suggest that the effect of RV pacing on LV strain is burden-dependent in TAVI patients. While correlations between chronic pacing and diastolic dysfunction have been reported [20,32], we did not observe significant differences between the two groups concerning diastolic function measurements (Table 2).

Our data support the hypothesis that chronic RV pacing can cause subclinical LV systolic dysfunction despite a beneficial LV afterload reduction effect from relieving aortic stenosis in TAVI patients. This effect is detectable by speckle tracking strain in the face of unchanged LVEF. Concerning the similar NYHA class distributions (Table 3), our findings suggest that chronic RV pacing-induced myocardial dysfunction is relatively mild at the 1-year follow-up time point. Therefore, sedentary status in our study cohorts and placebo effect of the TAVI procedure may have contributed to lack of difference in NYHA class at follow up in our study groups. Studies with longer-term follow-up are still warranted to determine whether the subclinical dysfunction will turn into clinical events in this patient population. Speckle tracking strain analysis on follow-up echocardiography studies in post-TAVI patients may allow identification of higher risk patients who need closer follow-up and who may benefit from timely biventricular device upgrade [33].

## 5. Limitations

This study is limited by its retrospective nature, and the cohort (2012–2019) may not reflect contemporary patient characteristics. While this is a single-institution study, the patient population was from all three different sites (MN, AZ, and FL). Detailed pacing configuration (e.g., RV lead position and RV stimulated QRS duration) was not available. HFH events could be missed if patients were hospitalized outside of our hospital. The study features a relatively short follow-up period, with a median of 354 days, which constrains our ability to assess the development of PiCM, which typically requires a longer timeframe to manifest. Speckled-tracking strain analysis was available in about 85% of patients of the entire cohort due to image quality or missing views. Additionally, ventricular rhythm (spontaneous or stimulated) at the time of the echocardiography study was not available, which could affect the validity of strain data. Furthermore, due to the possibility of the study being underpowered, we cannot exclude the likelihood of type II errors contributing to the negative results regarding clinical endpoints. We only reported individual diastolic function parameters as diastolic function grading was not consistently available across the cohort, and two different versions of diastolic function guidelines were used during the study period.

## 6. Conclusions

Patients who received chronic RV pacing after the TAVI procedure can develop subclinical LV dysfunction despite having preserved LVEF. This group of patients needs to be closely monitored for the development of heart failure or LV dysfunction.

## Figures and Tables

**Table 1:** Patient demographics and clinical data.

	Non-Pacing Group	Pacing Group	*p*-Value
	N = 147	N = 147	
***Patient Demographics***			
**Age**	81.0 ± 7.3	81.2 ± 7.6	0.898
**Sex (male)**	92 (62.6%)	82 (55.8%)	0.235
**Race (white)**	142 (96.6%)	142 (96.6%)	0.753
**STS risk score**	8.5 ± 5.9	8.5 ± 7.8	0.922
**Prior MI**	31 (21.1%)	31 (21.1%)	1.000
**Prior PCI**	47 (32.0%)	26 (17.7%)	0.005
**Prior CABG**	45 (30.6%)	55 (37.4%)	0.218
**Prior Stroke**	14 (9.5%)	16 (10.9%)	0.700
**Prior PAD**	75 (51.0%)	80 (54.4%)	0.559
**Current dialysis**	9 (6.1%)	8 (5.4%)	0.803
**Smoker**	5 (3.4%)	6 (4.1%)	0.759
**Hypertension**	124 (84.4%)	128 (87.1%)	0.505
**Diabetes**	60 (40.8%)	60 (40.8%)	1.000
**Afib/Aflutter**	54 (36.7%)	62 (42.2%)	0.340
**Pre-TAVR Conduction defect**	61 (41.5%)	83 (56.5%)	0.010
**Hgb (g/dL)**	12.1 ± 1.9	11.8 ± 1.9	0.095
**Creatinine (mg/dL)**	1.5 ± 1.5	1.4 ± 1.1	0.423
***Medications***			
**Aspirin**	121 (82.3%)	122 (83.0%)	0.999
**Beta blocker**	105 (71.4%)	103 (70.1%)	0.344
**ACE-I**	30 (20.4%)	26 (17.7%)	0.809
**ARB**	13 (8.8%)	11 (7.5%)	0.760
**Warfarin**	75 (51.0%)	89 (60.5%)	0.256
***ECG (baseline)***			
**Ventricular rate (bpm)**	70.3 ± 14.2	73.4 ± 14.9	0.051
**PR (ms)**	194.2 ± 44.9	187.5 ± 43.4	0.128
**QRS (ms)**	115.6 ± 28.0	111.3 ± 26.2	0.095
**QT (ms)**	427.8 ± 44.6	415.6 ± 41.8	0.015
**QTc (ms)**	456.5 ± 34.6	453.3 ± 34.1	0.175
**RBBB**	26 (17.7%)	44 (29.9%)	0.020
**LBBB**	13 (8.8%)	9 (6.1%)	0.506
**Variable AV Block**	2 (1.4%)	4 (2.7%)	0.680
**1st Degree AV Block**	27 (18.4%)	43 (29.3%)	0.040
**2nd Degree AV Block**	0 (0.0%)	1 (0.7%)	1.000
**Left Anterior Fascicular Block**	7 (4.8%)	22 (15.0%)	0.006
**Left Posterior Fascicular Block**	0 (0.0%)	5 (3.4%)	0.071
***Procedural Outcomes***			
**Perforation (with or without tamponade)**	1 (1.1%)	0 (0.0%)	0.802
**Unplanned Vascular Surgery/Intervention**	6 (6.7%)	0 (0.0%)	0.006
**Unplanned other Cardiac Surgery/Intervention**	1 (1.1%)	1 (0.7%)	0.706
**Post-TAVI mean AVG (mmHg)**	12.3 ± 6.4	10.9 ± 4.7	0.049
***Pacing Indication(s)***			
**AVB**	--	57 (39.9%)	--
**Complete AVB**	--	31 (21.7%)	--
**LBBB**	--	36 (25.2%)	--
**Sinus Node Dysfunction**	--	10 (7.0%)	--
**Bradycardia**	--	6 (4.2%)	--
**Bifascicular block**	--	2 (1.4%)	--
**RBBB**	--	1 (0.7%)	--
***Pacing Mode(s)***			
**DDD/DDDR**	--	123 (83.1%)	--
**VVI/VVIR**	--	21 (14.2%)	--
**Other**	--	4 (2.8%)	--

Continuous variables are expressed in mean ± SD, categorical variables are expressed in N(%). MI: myocardial infarction, PCI: percutaneous coronary intervention, CABG: coronary artery bypass graft, PAD: peripheral arterial diseases, Afib: atrial fibrillation, Aflutter: atrial flutter, TAVR: transcatheter aortic valve replacement, RBBB: right bundle branch block, LBBB: left bundle branch block.

**Table 2:** LV function and strain measurements.

	Non-Pacing Group			Pacing Group			Two-Group Comparison	
	N = 147			N = 147			Baseline	Follow-Up
	Baseline	Follow-Up	* *p*-Value	Baseline	Follow-Up	* *p*-Value	** *p*-Value	*** *p*-Value
**LVEF (%)**	53.8 ± 13.9	58.8 ± 11.2	0.001	56.7 ± 10.9	57.1 ± 13.0	0.775	0.455	0.308
**E/A ratio**	1.0 ± 0.7	1.1 ± 0.6	0.784	1.1 ± 0.6	0.9 ± 0.5	0.216	0.742	0.325
**Medial e’ (m/s)**	0.05 ± 0.01	0.06 ± 0.02	0.002	0.06 ± 0.1	0.05 ± 0.02	0.300	0.525	0.064
**Lateral e’ (m/s)**	0.08 ± 0.03	0.07 ± 0.02	<0.001	0.06 ± 0.02	0.06 ± 0.02	0.165	0.331	0.059
**Average E/e’ ratio**	16.2 ± 8.2	16.1 ± 9.9	0.846	18.7 ± 9.8	18.9 ± 8.8	0.539	0.128	0.162
**LV mass index (g/m^2^)**	128.0 ± 99.1	121.2 ± 30.5	0.280	119.7 ± 33.7	121.2 ± 33.8	0.555	0.375	0.998
**RVSP (mmHg)**	40.6 ± 11.8	41.5 ± 12.7	0.323	42.8 ± 14.9	43.6 ± 12.6	0.562	0.167	0.309
**LV global longitudinal strain (%)**	−14.9 ± 4.3	−18.8 ± 2.7	<0.0001	−13.6 ± 3.5	−12.7 ± 3.5	0.021	0.008	<0.0001
**LV Regional Apex (%)**	−18.5 ± 6.1	−23.2 ± 9.2	<0.0001	−17.1 ± 5.4	−12.9 ± 5.5	<0.0001	0.069	<0.0001
**LV Regional Mid (%)**	−14.6 ± 5.6	−18.7 ± 3.4	<0.0001	−13.2 ± 4.3	−12.7 ± 4.6	0.283	0.018	<0.0001
**LV Regional Basal (%)**	−12.5 ± 6.3	−13.0 ± 3.8	0.006	−10.8 ± 4.3	−12.9 ± 4.1	0.009	0.137	0.260

* Baseline vs. follow-up; ** non-pacing vs. pacing (at baseline); *** non-pacing vs. pacing (at follow-up). LV: left ventricular, EF: ejection fraction.

**Table 3:** Comparison of NYHA class at baseline and follow-up.

NYHA Class	Baseline		Follow-Up	
	Pacing	Non-Pacing	Pacing	Non-Pacing
I	8.0 (5.4%)	4.0 (2.7%)	108.0 (73.5%)	109.0 (74.1%)
II	32.0 (21.8%)	22.0 (15.0%)	34.0 (23.1%)	28.0 (19.0%)
III	94.0 (63.9%)	104.0 (70.7%)	3.0 (2.0%)	9.0 (6.1%)
IV	13.0 (8.8%)	17.0 (11.6%)	2.0 (1.4%)	1.0 (0.7%)
*p*-value	0.238		0.270	

## Data Availability

Data are contained within the article and Supplementary Materials.

## Supplementary Materials

The following supporting information can be downloaded at: https://www.mdpi.com/article/10.3390/jcm13154553/s1, Table S1: Characteristics used for propensity cohort match.

## Abbreviations

STS:	Society of Thoracic Surgeons
SAVR:	surgical aortic valve replacement
TAVI:	transcatheter aortic valve implantation
RV:	right ventricular
TTE:	transthoracic echocardiography
HFH:	heart failure hospitalization
PPM:	permanent pacemaker
ICD:	implantable cardioverter-defibrillator
DDD/DDDR:	pacing mode
VVI/VVIR:	pacing mode
