# Supplementary material for: Chronic Right Ventricular Pacing Post-Transcatheter Aortic Valve Replacement Attenuates the Benefit on Left Ventricular Function
Source: J Clin Med. 2024 Aug 4;13(15):4553. doi: 10.3390/jcm13154553 (PMC11313289; doi:10.3390/jcm13154553)
Supplement: Supplementary file 1 [file jcm-13-04553-s001.zip › jcm-2990798-supplementary.pdf]

Table S1. Characteristics used for propensity cohort match.

	Pacing group	Matched Cohort (non-pacing)	Initial Unmatched Cohort (non-pacing)
	n=147	n=147	n=903
Age	81.2 ±7.6	81.0 ±7.3	81.0 ± 8.7
Sex (Male)	82 (55.8%)	92 (62.6%)	519 (57.5%)
Race (White)	142 (96.6%)	142 (96.6%)	874 (96.8%)
STS risk Score	8.5 ±7.8	8.5 ±5.9	8.0 ± 5.2
